# Supplementary material for: Identification of photoperiod-induced specific miRNAs in the adrenal glands of Sunite sheep (Ovis aries)
Source: Front Vet Sci. 2022 Jul 22;9:888207. doi: 10.3389/fvets.2022.888207 (PMC9354845; doi:10.3389/fvets.2022.888207)
Supplement: Supplementary file 1 [file Data_Sheet_1.ZIP › Supplementary file/miRDeep2 file.zip!/miRDeep2 file/Know_Structure/SP42A1/oar-mir-199a.pdf]

miRBase precursor : oar-mir-199a
Total read count : 100593
oar-miR-199a-3p read count : 63276
remaining reads : 37317

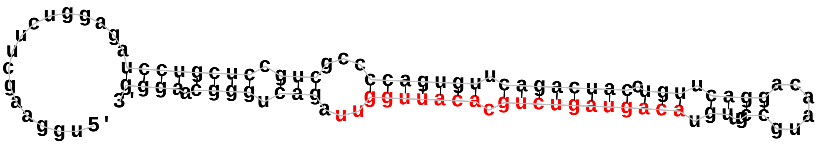

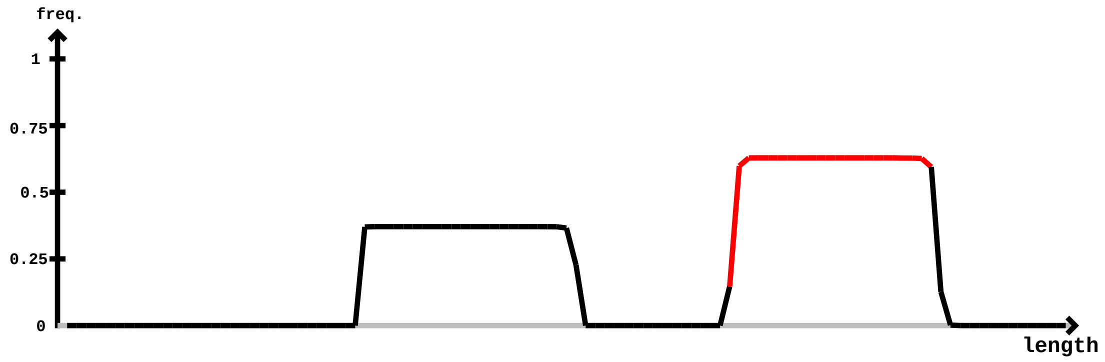

oar-miR-199a-3p

```
5'- uggaagcuucuggagauccugcuccgucgccccaguguucagacuaccuguucaggacaaugccguuguacaguagucugcacauugguuagacugggcaaggg -3'  exp
    .................(((((((.(((...(((((((.((((((((.(((.((((......))..)).))))))))))).)))))))...))).)))).))).  reads  mm  sample
    .............................ccccaguguucagacuaccuguu....................................................  3      0   seq
    .............................ccccaguguucagacuaccuguuc...................................................  1      0   seq
    ..............................cccaguguucagacuacc........................................................  1      0   seq
    ..............................cccaguguucagacuaccu.......................................................  22     0   seq
    ..............................cccaguguucagacuaccug......................................................  28     0   seq
    ..............................cccaguguucagacuaccugu.....................................................  480    0   seq
    ..............................cccaguguucagacuaccuguu....................................................  13917  0   seq
    ..............................cccaguguucagacuaccuguuc...................................................  22743  0   seq
    ...............................ccaguguucagacuaccuguu....................................................  41     0   seq
    ...............................ccaguguucagacuaccuguuc...................................................  71     0   seq
    ................................caguguucagacuaccuguu....................................................  5      0   seq
    ................................caguguucagacuaccuguuc...................................................  3      0   seq
    .................................aguguucagacuaccuguu....................................................  2      0   seq
    ....................................................................uacaguagucugcacau...................  1      0   seq
    ....................................................................uacaguagucugcacauu..................  24     0   seq
    ....................................................................uacaguagucugcacauug.................  12     0   seq
    ....................................................................uacaguagucugcacauugg................  73     0   seq
    ....................................................................uacaguagucugcacauuggu...............  1638   0   seq
    ....................................................................uacaguagucugcacauugguu..............  11984  0   seq
    ....................................................................uacaguagucugcacauugguua.............  1023   0   seq
    ....................................................................uacaguagucugcacauugguuag............  11     0   seq
    ....................................................................uacaguagucugcacauugguuaga...........  1      0   seq
    .....................................................................acaguagucugcacauu..................  58     0   seq
    .....................................................................acaAuagucugcacauu..................  1      1   seq
    .....................................................................acaguagucugcacauug.................  10     0   seq
    .....................................................................acaguagucugcacauugg................  64     0   seq
    .....................................................................acaguagucugcacauuggu...............  1424   0   seq
    .....................................................................acaguagucugcacauugguu..............  33000  0   seq
    .....................................................................acaguagucugcacauugguua.............  10747  0   seq
    .....................................................................acaguagucugcacauugguuag............  126    0   seq
    .....................................................................acaguagucugcacauugguuaga...........  10     0   seq
    ......................................................................caguagucugcacauug.................  1      0   seq
    ......................................................................caguagucugcacauugg................  7      0   seq
    ......................................................................caguagucugcacauuggu...............  130    0   seq
    ......................................................................caguagucugcacauugguu..............  2111   0   seq
```

oar-miR-199a-3p

```
uggaagcuucuggagauccugcuccgucgccccaguguucagacuaccuguucaggacaaugccguuguacaguagucugcacauugguuagacugggcaaggg
......................................................................caguagucugcacauugguua.............    776    0    seq
......................................................................caguagucugcacauugguuag............    20     0    seq
......................................................................caguagucugcacauugguuaga...........    1      0    seq
.......................................................................aguagucugcacauugguu..............    12     0    seq
.......................................................................aguagucugcacauugguua.............    10     0    seq
.......................................................................aguagucugcacauugguuag............    1      0    seq
```
